# The research domain criteria (RDoC) project as a biological framework for research on mental disorders

**DOI:** 10.1192/j.eurpsy.2025.69

**Published:** 2025-08-26

**Authors:** C. Sanislow

**Affiliations:** Wesleyan University, Essex, United States

## Abstract

**Abstract:**

The U.S. National Institute of Mental Health (NIMH) Research Domain Criteria (RDoC) offers a framework to facilitate clinical research for psychopathology. Unique features include an emphasis on transdiagnostic dimensions spanning normal and abnormal ranges of function, novel independent variables (mechanisms, not diagnoses), and the integration of different kinds of observations (neural, psychological, self-report). In this talk, principles and pragmatics of RDoC approach are illustrated with examples from RDoC-framed clinical research, and the clinical significance is discussed.

**Disclosure of Interest:**

None Declared

